# *KRAS* Mutations and Primary Resistance of Lung Adenocarcinomas to Gefitinib or Erlotinib

**DOI:** 10.1371/journal.pmed.0020017

**Published:** 2005-01-25

**Authors:** William Pao, Theresa Y Wang, Gregory J Riely, Vincent A Miller, Qiulu Pan, Marc Ladanyi, Maureen F Zakowski, Robert T Heelan, Mark G Kris, Harold E Varmus

**Affiliations:** **1**Program in Cancer Biology and Genetics, Memorial Sloan-Kettering Cancer CenterNew York, New YorkUnited States of America; **2**Department of Medicine, Memorial Sloan-Kettering Cancer CenterNew York, New YorkUnited States of America; **3**Department of Pathology, Memorial Sloan-Kettering Cancer CenterNew York, New YorkUnited States of America; **4**Department of Radiology, Memorial Sloan-Kettering Cancer CenterNew York, New YorkUnited States of America; MD Anderson Cancer CenterUnited States of America

## Abstract

**Background:**

Somatic mutations in the gene for the epidermal growth factor receptor (EGFR) are found in adenocarcinomas of the lung and are associated with sensitivity to the kinase inhibitors gefitinib (Iressa) and erlotinib (Tarceva). Lung adenocarcinomas also harbor activating mutations in the downstream GTPase, KRAS, and mutations in *EGFR* and *KRAS* appear to be mutually exclusive.

**Methods and Findings:**

We sought to determine whether mutations in *KRAS* could be used to further enhance prediction of response to gefitinib or erlotinib. We screened 60 lung adenocarcinomas defined as sensitive or refractory to gefitinib or erlotinib for mutations in *EGFR* and *KRAS*. We show that mutations in *KRAS* are associated with a lack of sensitivity to either drug.

**Conclusion:**

Our results suggest that treatment decisions regarding use of these kinase inhibitors might be improved by determining the mutational status of both *EGFR* and *KRAS*.

## Abstract 2

Mutational analysis of the *KRAS* gene in lung cancer patients treated with two different kinase inhibitors suggests that tumors with KRAS mutations do not respond to these drugs

## Introduction

Genes of the *ERBB* family encode receptor tyrosine kinases that mediate cellular responses to growth signals. Somatic mutations in the tyrosine kinase domains of two *ERBB* genes, *epidermal growth factor receptor (EGFR)* and *HER2,* have been found in a proportion of lung adenocarcinomas [1,2,3,4]. For *EGFR,* mutations are associated with sensitivity to the small-molecule kinase inhibitors gefitinib (Iressa) [1,2,3] and erlotinib (Tarceva) [3].

ERBB signaling pathways include downstream GTPases encoded by *RAS* genes. Some 15%–30% of lung adenocarcinomas contain activating mutations in the *RAS* family member *KRAS*. These mutations are most frequently found in codons 12 and 13 in exon 2 [5,6], and may be associated with unfavorable outcomes [7]. Interestingly, *EGFR* and *KRAS* mutations are rarely found in the same tumors, suggesting that they have functionally equivalent roles in lung tumorigenesis ([8]; M. Meyerson, personal communication). Furthermore, *EGFR* mutations are common in tumors from patients who have smoked less than 100 cigarettes in their lifetimes (“never smokers”) [3], while *KRAS* mutations more commonly occur in individuals with a history of substantial cigarette use [9].

We sought to determine whether *KRAS* mutations could also be used to predict primary sensitivity or resistance to gefitinib or erlotinib. We systematically evaluated 60 lung adenocarcinomas from patients with known responses to either of these drugs for the presence of mutations in *EGFR* (exons 18 through 21) and *KRAS2* (exon 2). Here, we show that mutations in *KRAS* are associated with primary resistance to single-agent gefitinib or erlotinib. Our results suggest that a determination of mutational status for both *EGFR* and *KRAS* may help define which patients are likely to benefit from receiving gefitinib or erlotinib.

## Methods

### Tissue Procurement

Tumor specimens were obtained through protocols approved by the institutional review board of Memorial Sloan-Kettering Cancer Center, as previously described [3] (see Protocols S1–S3). Tumor material, obtained from patients prior to kinase inhibitor treatment for lung cancer, was collected retrospectively for patients on gefitinib, who received 250 mg or 500 mg orally once daily (*n* = 24), and prospectively for patients on erlotinib, who received 150 mg orally once daily (*n* = 36). The latter cohort of patients was part of a clinical trial of erlotinib for patients with bronchioloalveolar carcinoma. The analysis presented here includes specimens we previously reported on (*n* = 17 for gefitinib and *n* = 17 for erlotinib) [3].

All specimens were reviewed by a single reference pathologist (M. F. Z.). Imaging studies were assessed by a single reference radiologist (R. T. H.), who graded responses according to Response Evaluation Criteria in Solid Tumors (RECIST) [10]. Both observers were blinded to patient outcomes.

Eight of nine patients with tumors sensitive to gefitinib had objective partial responses as defined by RECIST, i.e., at least a 30% decrease in the sum of the longest diameters of target lesions, taking as reference the sum measured at baseline. The ninth patient had marked clinical improvement, as ascertained by two independent reviewing physicians and manifested by lessened dyspnea and cancer-related pain. However, this individual had radiographic lesions (pleural and bone metastases) that were deemed nonmeasurable by RECIST criteria. As erlotinib-treated patients were all in a clinical trial, all had disease measurable using RECIST guidelines. For both drugs in this study, tumors were considered refractory if they did not undergo sufficient shrinkage to qualify for partial response. This definition includes patients whose “best overall response” was either progression of disease (*n* = 26) or stable disease (*n* = 12) as defined by RECIST. No patients had a complete response.

### Mutational Analyses of *EGFR* and *KRAS* in Lung Tumors

Genomic DNA was extracted from tumors embedded in paraffin blocks, except for tumor 109T, which was a fresh-frozen tumor specimen. Primers for *EGFR* analyses (exons 18–21) were as published [3]. For *KRAS* analyses, the following nested primer sets for exon 2 were used: huKRAS2 ex2F, 5′- GAATGGTCCTGCACCAGTAA-3′; huKRAS2 ex2R, 5′- GTGTGACATGTTCTAATATAGTCA-3′; huKRAS2 ex2Fint, 5′- GTCCTGCACCAGTAATATGC-3′; and huKRAS2 ex2Rint, 5′- ATGTTCTAATATAGTCACATTTTC-3′.

For both *EGFR* and *KRAS,* PCR was performed using the HotStarTaq Master Mix Kit (Qiagen, Valencia, California, United States), as per manufacturer's instructions. Use of this method often obviated the need for nested PCR sets. All sequencing reactions were performed in both forward and reverse directions, and all mutations were confirmed by PCR amplification of an independent DNA isolate.

In 12 cases, exon 19 deletions were also studied by length analysis of fluorescently labeled PCR products on a capillary electrophoresis device, using the following primers: *EGFR*-Ex19-FWD1, 5′- GCACCATCTCACAATTGCCAGTTA-3′, and *EGFR*-Ex19-REV1, 5′-Fam- AAAAGGTGGGCCTGAGGTTCA-3′. Using serial dilutions of DNA from the H1650 non-small-cell lung cancer cell line (exon 19 deletion-positive [11]), this assay detects the mutant allele when H1650 DNA comprises 6% or more of the total DNA tested, compared to a sensitivity of 12% for direct sequencing. These same cases were also screened for the exon 21 L858R mutation by a PCR–restriction fragment length polymorphism assay, based on a new Sau96I restriction site created by the L858R mutation (2,573T→G). The Sau96I-digested fluorescently labeled PCR products were analyzed by capillary electrophoresis, and the following primers were used: *EGFR*-Ex21-FWD1, 5′- CCTCACAGCAGGGTCTTCTCTGT-3′, and *EGFR*-Ex21-REV1, 5′-Fam- TCAGGAAAATGCTGGCTGACCTA-3′. Using serial dilutions of DNA from the H1975 cell line (L858R-positive [11]), this assay detects the mutant allele when H1975 DNA comprises 3% or more of the total DNA tested, compared to a sensitivity of 6% for direct sequencing (Q. Pan, W. Pao, and M. Ladanyi, unpublished data).

### Statistics

Fisher's Exact Test was used to calculate *p-*values, and confidence intervals were calculated using *Statistics with Confidence* software [12].

## Results

We identified 60 lung adenocarcinomas from individual patients with tumors shown to be sensitive or refractory to single-agent gefitinib or erlotinib and evaluated these tumors for mutations in *EGFR* and *KRAS*. Collectively, nine of 38 (24%) tumors refractory to either kinase inhibitor had *KRAS* mutations, while zero of 21 (0%) drug-sensitive tumors had such mutations (*p* = 0.02) (Table 1). The 95% confidence intervals (CIs) for these observations are 13%–39% and 0%–16%, respectively. Conversely, 17 of 22 (77%) tumors sensitive to either kinase inhibitor had *EGFR* mutations, in contrast to zero of 38 (0%) drug-resistant tumors (*p* = 6.8 × 10^−11^). The 95% CIs for these observed response rates are 57%–90% and 0%–9%, respectively. All 17 tumors with *EGFR* mutations responded to gefitinib or erlotinib, while all nine tumors with *KRAS* mutations did not (*p* = 3.2 × 10^−7^).

**Table 1:**
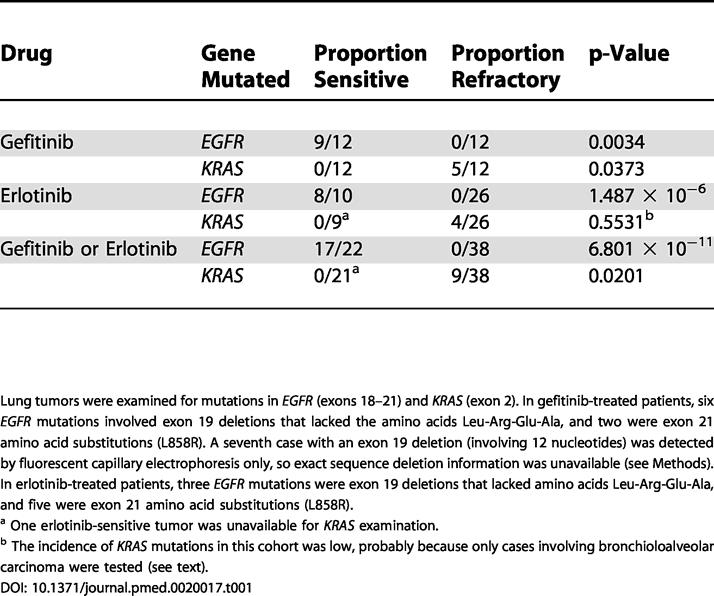
*EGFR* and *KRAS* Mutation Status in Lung Adenocarcinomas Sensitive or Refractory to Gefitinib or Erlotinib

Lung tumors were examined for mutations in *EGFR* (exons 18–21) and *KRAS* (exon 2). In gefitinib-treated patients, six *EGFR* mutations involved exon 19 deletions that lacked the amino acids Leu-Arg-Glu-Ala, and two were exon 21 amino acid substitutions (L858R). A seventh case with an exon 19 deletion (involving 12 nucleotides) was detected by fluorescent capillary electrophoresis only, so exact sequence deletion information was unavailable (see Methods). In erlotinib-treated patients, three *EGFR* mutations were exon 19 deletions that lacked amino acids Leu-Arg-Glu-Ala, and five were exon 21 amino acid substitutions (L858R)

^a^ One erlotinib-sensitive tumor was unavailable for *KRAS* examination

^b^ The incidence of *KRAS* mutations in this cohort was low, probably because only cases involving bronchioloalveolar carcinoma were tested (see text)

**Figure 1:**
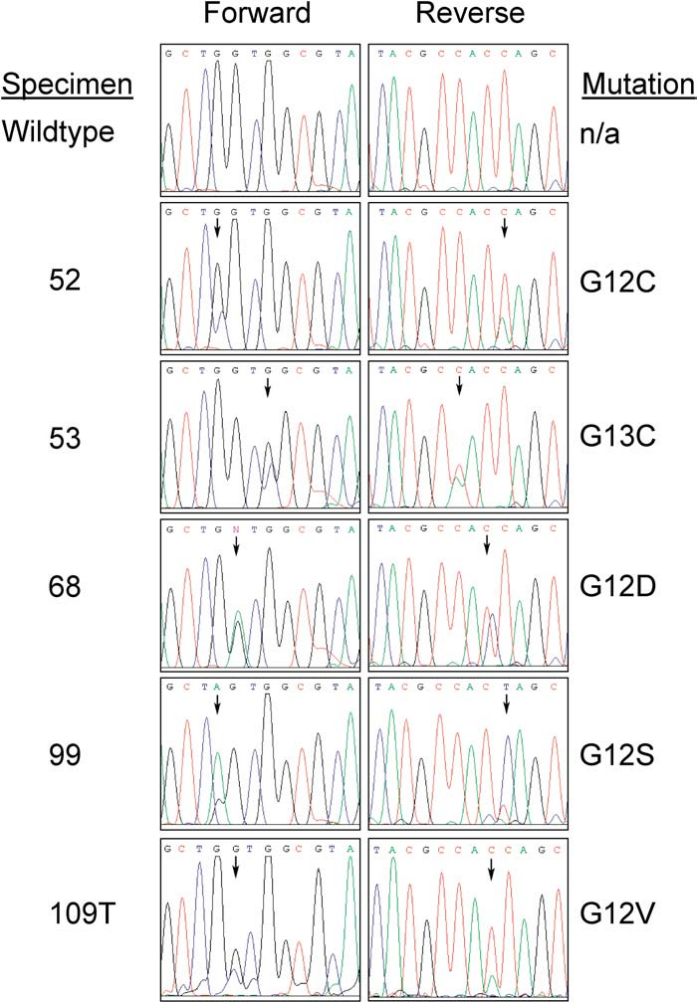
Sequence Chromatograms Displaying the Types of *KRAS* Mutations Found in This Study

Correlation of *EGFR* and *KRAS* mutational status with drug and treatment response is detailed in Table 1. The spectrum of *KRAS* mutations is shown in Figure 1 and Table 2. Results with gefitinib and erlotinib were similar overall. However, the incidence of *KRAS* mutations in the patients treated with erlotinib was low, probably because of the fact that all patients treated with this drug had bronchioloalveolar carcinoma, which rarely has *RAS* mutations [13]. Alternatively, our analyses involving only exon 2 of *KRAS2* may have missed some *RAS* mutations. However, in our analysis of the exonic regions encoding the first 100 amino acids of *KRAS* in 110 surgically resected early-stage non-small-cell lung cancers, we have found 18 mutations, and all were in either codon 12 or codon 13, encoded by exon 2 (W. Pao, R. Wilson, H. Varmus, unpublished data). Another possibility is that the erlotinib-treated tumors have mutations in other *RAS* genes, since a minority of *RAS* mutations in lung cancer have been reported to occur in *N-* or *HRAS* [5,6].

**Table 2:**
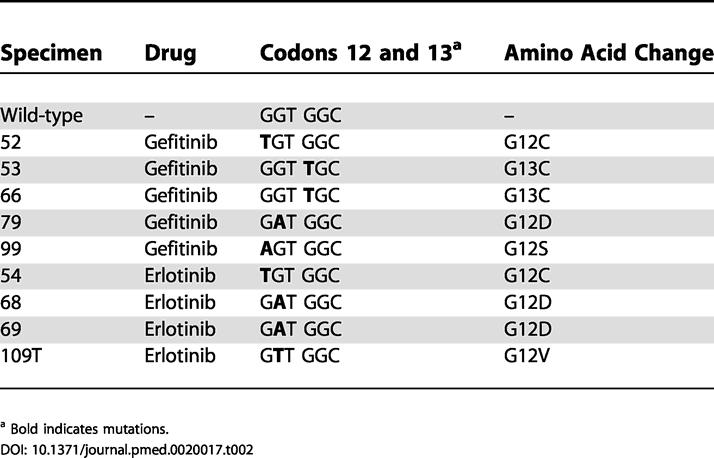
*KRAS* Exon 2 Mutations Found in Non-Small-Cell Lung Cancers Refractory to Treatment with Gefitinib or Erlotinib

^a^ Bold indicates mutations

## Discussion

These results have important clinical implications. First, they extend previous data from our group and others showing that lung adenocarcinomas containing *EGFR* mutations are associated with sensitivity to gefitinib or erlotinib (17 of 17 in this series; 100% observed response rate; 95% CI, 82%–100%). Second, these data show that tumors with *KRAS* exon 2 mutations (*n* = 9) are associated with a lack of response to these kinase inhibitors (0% observed response rate; 95% CI, 0%–30%). Third, no drug-sensitive tumors had *KRAS* exon 2 mutations (*n* = 21). Whether *KRAS* mutational status can be used to predict responses to gefitinib or erlotinib in patients whose tumors have wild-type *EGFR* sequence is still under investigation: our analysis comparing response rates for tumors with neither *EGFR* nor *KRAS* mutations versus tumors with wild-type *EGFR* but mutated *KRAS* does not reach statistical significance (five of 22 versus zero of nine; *p* = 0.29). Nevertheless, these findings suggest that patients whose lung adenocarcinomas have *KRAS* mutations will not experience significant tumor regression with either drug.

The incidence of *EGFR* mutations in tumors responsive to EGFR kinase inhibitors has varied from 71% to 100% ([1,2,3] and this paper). Thus, at this point, patients whose tumors test negative for *EGFR* mutations should not necessarily be precluded from treatment with either gefitinib or erlotinib. Data presented here suggest that clinical decisions regarding the use of these agents in patients with lung adenocarcinomas might be improved in the future by pre-treatment mutational profiling of both *EGFR* and *KRAS*. These findings warrant validation in large prospective trials using standardized mutation detection techniques.

### Supporting Information

Protocol S1 Preclinical Studies of Blood, Urine, Bone Marrow, and Tissues Collected from Patients with Thoracic Malignancies (32 KB PDF).

Protocol S2 Multicenter Phase II Trial of OSI-774 (Erlotinib, Tarceva) in Patients with Advanced Bronchioloalveolar Cell Lung Cancer (1.9 MB PDF).

Protocol S3 Protocol Approval Letters (60 KB PDF).

### Accession Numbers

The LocusLink (http://www.ncbi.nlm.nih.gov/LocusLink/) accession number for the *KRAS2* sequence discussed in this paper is 3845; the GenBank (http://www.ncbi.nlm.nih.gov/Genbank/) accession number for the *KRAS2* sequence discussed in this paper is NT_009714.16.

**Patient Summary**

**Background**

Two drugs, gefitinib (Iressa) and erlotinib (Tarceva), have been developed that can make lung cancers smaller in some patients. The drugs work by blocking the effect of a molecule called the epidermal growth factor receptor (EGFR), which relays instructions to cells to grow and divide. Recently, researchers found that these drugs most effectively shrink tumors that have acquired abnormal variations (mutations) in the *EGFR* gene. These mutations somehow allow tumor cells to escape normal safety mechanisms that keep cells from growing out of control. Some lung cancers also have mutations in another gene called *KRAS*. Interestingly, *KRAS* mutations and *EGFR* mutations are rarely ever found in the same tumor.

**Why Was This Study Done?**

Unfortunately, *EGFR* mutations are only found in a minority of patients with lung cancer. This means that gefitinib or erlotinib might be given to a lot of patients who may not benefit from this treatment. Ideally, the drugs would be given only to patients who we know will benefit from them. This study examined whether studying the *KRAS* gene (to see if it had a mutation) could help predict which patients had tumors that would respond well to the drugs.

**What Did the Researchers Do?**

They took 60 lung cancer samples from patients who had been treated with one of the drugs and either responded (that is, their tumors shrunk in size) or not, and tested whether the tumors had normal or abnormal *KRAS*.

**What Did They Find?**

Tumors that got significantly smaller while treated with gefitinib or erlotinib (a total of 22) had a normal *KRAS* gene*.* Most of these tumors had *EGFR* mutations. Conversely, tumors that had abnormal *KRAS* (a total of nine) did not shrink while treated with gefitinib or erlotinib.

**What Does This Mean?**

Both gefitinib and erlotinib are expensive and have side effects. Testing for *EGFR* and *KRAS* mutations is relatively straightforward, and one could test for abnormalities in both genes first and then decide which patients should be treated with either of the two drugs.

**What Next?**

Before doing *EGFR* and *KRAS* tests on a routine basis and taking the results into account when making a decision about who should be treated with gefitinib or erlotinib, larger studies need to be done to see whether the results reported here hold up.

**More Information Online**

US Food and Drug Administration information page on Iressa: http://www.fda.gov/cder/drug/infopage/iressa/iressaQ&A.htm

Cancer Research UK information page about erlotinib: http://www.cancerhelp.org.uk/help/default.asp?page=10296

## Abbreviations

CI: confidence interval
EGFR: epidermal growth factor receptor
RECIST: Response Evaluation Criteria in Solid Tumors
