# Supplementary material for: KRAS Mutations and Primary Resistance of Lung Adenocarcinomas to Gefitinib or Erlotinib
Source: PLoS Med. 2005 Jan 25;2(1):e17. doi: 10.1371/journal.pmed.0020017 (PMC545207; doi:10.1371/journal.pmed.0020017)
Supplement: Protocol S1 — (32 KB PDF). [file pmed.0020017.sd001.pdf]

THORACIC ONCOLOGY SERVICE - DEPARTMENT OF MEDICINE

PRECLINICAL STUDIES OF BLOOD, URINE, BONE MARROW, AND TISSUES COLLECTED FROM PATIENTS WITH THORACIC MALIGANCIES

	Page
1.0 OBJECTIVE	2
2.0 BACKGROUND	2
3.0 PATIENT ELIGIBILITY	3
4.0 PLAN	3
5.0 CONFIDENTIALITY	4
6.0 CONSENT PROCEDURES	5
7.0 FACILITIES	5
8.0 REFERENCES	6

APPENDIX A: Informed Consent Form for Bone Marrow
APPENDIX B: Informed Consent Form for Blood and Urine Samples
APPENDIX C: Informed Consent Form for Tissue Samples

Principal Investigator:	Vincent Miller, MD*
Co-Investigators:	
Thoracic Medicine	Mark Kris, MD*
	Lee Krug, MD*
	Herbert Oettgen, MD*
	Jorge Gomez, MD*
	Christopher G. Azzoli, MD*
	William Pao, MD*
	Naiyer A. Rizvi, MD*
	Anne C. Chiang, MD*
	Katerina Politi, PhD
Thoracic Surgery	Manjit Bains, MD*
	Robert Downey, MD*
	Raja Flores, MD*
	Bernard Park, MD*
	Valerie Rusch, MD*
	Bhuvanesh Singh, MD*
	Nabil Rizk, MD*
Department of Pathology	Carlos Cordon-Cardo, MD PhD
	Ronald Ghossein, MD
	Maureen Zakowski, MD
Clinical Immunology	Alan Houghton, MD
Neurology	Jerome Posner, MD
	Lauren Abrey, MD*

* Investigators eligible to obtain informed consent
Memorial Sloan Kettering Cancer Center
1275 York Avenue
New York, NY 10021

(212) 639 - 5126

1. OBJECTIVE

To study and store blood, urine, bone marrow, tissues and cell specimens from patients with thoracic malignancies (lung neoplasms, mesotheliomas, thymic malignancies) which will be used in current and subsequent immunological, biochemical, and molecular genetic analyses. These data will be integrated and correlated with current computer databases on these patients.

2. BACKGROUND

The American Cancer Society estimates that 146,000 Americans will die of lung cancer in 1992.' Lung cancer has always been the leading cause of death from cancer in American men and has now overtaken breast cancer as the leading cause of cancer death in women. The understanding of the biology of lung cancer will have direct applications in the treatment of this disease[2]. Aspects which have been explored include mechanisms of resistance [3] identification of active chemotherapy agents[4], phenotypic and functional characterization of lung cancer cell lines [5,6], and identification of potential targets for immune therapy[7,8]. Multiple collaborative studies investigating the biology of lung cancers are underway at Memorial Sloan Kettering Cancer Center. Many of these studies require fresh, operative tumor tissue for subsequent immunohistochemical, protein, DNA, and RNA assays. All of these studies require correlation of the experimental data with the clinical information to provide useful prognostic and therapeutic information. It is planned that laboratory studies will be integrated with the Clinical Research DataBase (CRDB) for non-small cell lung cancer[9], mesothelioma, small cell lung cancer and thymic malignancies including thymoma and thymic carcinoma.

3. PATIENT ELIGIBILITY

To obtain bone marrow or tissue specimens, patients must be undergoing a diagnostic or therapeutic procedure as part of their standard care, during which pathologic or cytologic material will be obtained. Patients having peripheral blood specimens collected will also be eligible.

The bone marrow or tissue specimens must be large enough to allow routine pathological analysis, with the specimen for the tumor bank taken from residual tissue that would otherwise be discarded.

4. PLAN

Our goal is to accrue a total of 700 patients to this protocol. In order to perform certain molecular genetic studies, tumor must be obtained in the operating room and placed immediately in liquid nitrogen. The following plan requires procurement from the operating room with coordination between both surgeon and pathologist to ensure optimal tumor specimens with minimal inconvenience and expense.

For each specimen, a portion will be sent to pathology for diagnostic confirmation. The residual tissue which has been resected and would otherwise be discarded would be utilized for the studies listed below. These tissues will be distributed under the direction of the tumor procurement service. In addition, when possible, a small portion of normal, control tissue would be obtained from the adjacent margins of the tumor and banked in a similar fashion. The collection of a tissue and serum is planned, so as to permit the performance of the described studies. The following is a general guide to procedures for tissue processing:

DNA and RNA Studies

1. The tissue is minced into 0.5 x 0.5 x 0.5 cm pieces and placed into individual plastic freezer bags.
2. The bags are appropriately labeled and placed into liquid nitrogen.
3. Final storage at -70° C.

Immunohistochemical Studies

1. The tissue is kept in saline on ice.
2. 0.5 x 0.5 x 0.5 cm pieces are placed in cryomolds and covered with OCT embedding medium.
3. The tissues are frozen in isopentane or N-hexane which has been cooled in liquid mtrogen.
4. Final storage at -70° C.

Protein and Glycolipids

Tissues will be frozen at -70° C.

Establishment of Cell Lines

The tissue is kept sterile and transported to the laboratory for further processing while fresh.

Blood Specimens

Serum and/or plasma specimens will be kept on ice and centrifuged as soon as possible. Specimens will be stored at -70^{0}C until the time of analysis.

Coding

In order to integrate laboratory specimens with the other clinical databases, specimens will be labeled with the patient’s medical record number.

5. CONFIDENTIALITY

As mentioned above, laboratory analyses must be correlated with the clinical databases, so all specimens require the presence of the medical record number.

6. CONSENT PROCEDURES

If experimental therapy is to be based on these data, or if bone marrow studies or biopsies are being obtained solely for research purposes a separate consent and IRB protocol will be required. Consent will be obtained from patients undergoing analysis or removal of bone marrow, blood, urine, and/or tissue samples as part of their routine care.

6.1 RESEARCH AUTHORIZATION

Procedures for obtaining Research Authorization: Before any protocol-specific procedures are carried out, investigators and/or designated staff will fully explain the details of the protocol, study procedures, and the aspects of patient privacy concerning research specific information. In addition to signing the IRB Informed Consent, all patients must sign the Research Authorization component of the informed consent form. The Research Authorization requires a separate signature from the patient. The original signed documents will become part of the patient's medical record, and each patient will receive a copy of the signed documents.

7. FACILITIES

The tumors will be banked by the Laboratory of Solid Tumor Immunology and The Laboratory of Molecular Medicine in their -70 ^{0}C Revco freezer. An alarm system is in place in the case of system failure.

8. PROTECTION OF HUMAN SUBJECTS

8.1 It is the responsibility of the Research Staff to ensure that Memorial Sloan-Kettering Cancer Center has on file a written acknowledgment of receipt by the subject of the Center's Notice of Privacy Practices. If the subject has not already done so, he/she must sign such an acknowledgment before participating in this study.

MSKCC's Privacy Office may allow the use and disclosure of protected health information pursuant to a completed and signed Research Authorization form. The use and disclosure of protected health information will be limited to the individuals described in the Research Authorization form. A Research Authorization form must be completed by the Principal Investigator and approved by the IRB and Privacy Board.

9. SUBJECT REGISTRATION

The following person(s) can obtain informed consent: Dr. Mark Kris, Dr. Lee Krug, Dr. Jorge Gomez, Dr. Christopher G. Azzoli, Dr. Vincent Miller, Dr. Naiyer A. Rizvi, Dr. Herbert Oettgen, Dr. William Pao, Dr. Anne C. Chiang, Dr. Manjit Bains, Dr.

Manjit Bains, Dr. Raja Flores, Dr. Bernard Park, Dr. Valerie Rusch, Dr. Buvanesh Singh, Dr. Nabil Rizk, Dr. Lauren Abrey.

Confirm with electronic medical record that the patient has signed the Notice of Privacy Practice. This must be obtained before the eligibility confirmation and obtaining of the research informed consent.

Confirm eligibility as defined in the section entitled Criteria for Patient/Subject Eligibility.

Obtain written informed consent, by following procedures defined in section entitled Informed Consent Procedures.

All patients must be registered through the Department of Medicine's (CTO) registration system at Memorial Sloan-Kettering Cancer Center. The CTO registry is available Monday through Friday from 8:15am - 5:00pm EST at 123-2150 (646/227-2150 from outside MSKCC). The last page of the signed consent form, the signature page of the Research Authorization, and a completed Eligibility Checklist must be faxed to the CTO registry at the time of registration. The fax number is 212-557-0786.

During the telephone registration process registerring individuals will be required to answer specific eligibility questions and provide the following information:

Registering Individual	[Last, First Name]
Notice of Privacy Status	[Yes, No]
Research Authorization	[Date]
MSKCC IRB Protocol#	
Attending of Record (if applicable)	[Last, First Name]
Consenting Professional	[Last, First Name]
Informed Consent Date	
Patient's Full Name	[Last, First Name]
Patient MRN	

10. REFERENCES

1. Boring CC, Squires TS, Heath CW, Jr. Cancer statistics 1992. Ca-A Cancer Journal for Clinicians. 1992, 42(1): 19-38.

2. Viallet J, Minna JD. Dominent oncogenes and tumor suppressor genes in the pathogenesis of lung cancer. Am. J. Respir. Cell Mol. Biol. 1990; 2: 225-232.

3. de Vries EGE, Meijer C, Timmer-Boscha H, et al. Resistance Mechanisms in three human small cell lung cancer cell lines established from one patient during clinical follow-up. Cancer Research 1989; 49: 4175-4178.

4. Carmichael J, Mitchell JB, DeGraff WG, et al. Chemosensitivity testing of human lung cancer cell lines using the MTT assay. Br. J. Cancer. 1988; *57(6):* 540-7.

5. Berendsen HH, de Leij L, de Vries EGE, et al. Characterization of three small cell lung cancer cell lines established from one patient during longitudinal follow-up. Cancer Research 1988; 48: 6891-6899.

6. Bepler G, Jacques G, Meumann K, et al. Establishment, growth properties, and morphological characteristics of permanent human small cell lung cancer cell lines. J. Cancer Res. ClinOncol. 1987; 113:31-40.

7. Brezicka FT, Olling S, Nilsson 0, et al. lmmunohistological detection of fucosyl-Gm_1 ganglioside in human lung cancer and normal tissues with monoclonal antibodies. Cancer Research. 1989; 49:1300-1305.

8. Lebacq-Verheyden AM, Neirynck A, Ravoet Am, et al. Monoclonal antibodies for the *in vitro* detection of small cell lung cancer metastases in human bone marrow. Eur. J. Clin. Oncol. 1988; 24 (2):137-145.

9. O'Connell JP, Kris MG, Gralla RJ, et al. Frequency and prognostic importance of pretreatment clinical characteristics in patients with advanced non-small cell lung cancer treated with combination chemotherapy. J Clin Oncol 1986-1 4 (11): 1604-14
