# Supplementary material for: KRAS Mutations and Primary Resistance of Lung Adenocarcinomas to Gefitinib or Erlotinib
Source: PLoS Med. 2005 Jan 25;2(1):e17. doi: 10.1371/journal.pmed.0020017 (PMC545207; doi:10.1371/journal.pmed.0020017)
Supplement: Protocol S2 — (1.9 MB PDF). [file pmed.0020017.sd002.pdf]

IRB#: 02-010A(8)

MULTICENTER PHASE II TRIAL OF OSI-774 (ERLOTINIB, TARCEVA™) IN PATIENTS WITH ADVANCED BRONCHIOLOALVEOLAR CELL LUNG CANCER

THERAPEUTIC/DIAGNOSTIC PROTOCOL

Principal Investigator:
Vincent A. Miller, M.D.
Medicine: Thoracic Oncology

Investigators:
Jorge Gomez, M.D.
Medicine: Thoracic Oncology
Mark G. Kris, M.D.
Medicine: Thoracic Oncology
Lee M. Krug, M.D.
Medicine: Thoracic Oncology
Herbert F. Oettgen, M.D.
Medicine: Thoracic Oncology
Christopher G. Azzoli, M.D.
Medicine: Thoracic Oncology
William Pao, M.D.
Medicine: Thoracic Oncology
Naiyer A. Rizvi, M.D.
Medicine: Thoracic Oncology
Maureen Zakowski, M.D.
Pathology: Surgical Pathology
Robert Heelan, M.D.
Radiology: Radiology
Mark Moasser, M.D.
Medicine: Breast Cancer Medicine
Bhuvanesh Singh, M.D.
Surgery: Head and Neck
Francis M. Sirotnak, PhD
Pathology: Molecular Pathology
Leslie Tyson, NP
Barbara Pizzo, RN
Megan Dunne,NP

Consenting Professionals:
Jorge Gomez, M.D.
Mark G. Kris, M.D
Lee M. Krug, M.D.
Vincent A. Miller, M.D.
Herbert Oettgen, M.D.
Christopher G. Azzoli, M.D.
William Pao, M.D.
Naiyer Rizvi, M.D.

Vanderbilt University Medical Center/ Ingram Cancer Center—Collaborating Institution

**Co-Principal Investigator:**	David Johnson, M.D.
**Investigators:**	Alan Sandler, M.D.<br>Jeffrey Sosman, M.D.<br>Kenneth Hande, M.D.<br>Kenneth Wyman, M.D.<br>Thao Dang, M.D.<br>David Carbone, Ph.D.,M.D.

University of Texas, MD Anderson Cancer Center – Collaborating Institution

**Principal Investigator:**	Roy S. Herbst, M.D., Ph.D.
**Investigators:**	Anne S Tsao, M.D.<br>George Blumenchein, Jr. , M.D.<br>Bonnie Glisson, M.D.<br>Frank Fossella, M.D.<br>Waun Ki Hong, M.D.<br>Charles Lu, M.D.<br>Yun Oh, M.D.<br>Vassiliki Papadimitrakopoulou, M.D.<br>Kathering Pisters, M.D.<br>Hai Tran, Pharm. D.<br>Ralph Zinner, M.D.<br>Jonathan Kurie, M.D.<br>Merril Kies, M.D.<br>Edward Kim, M.D.

Dana-Farber Cancer Institute – Collaborating Institution

**Principal Investigator:**	Michael Rabin, M.D.
**Investigators:**	Panos Fidias, M.D.<br>Sarada Gurubhagavatula, M.D.<br>John Heymach, M.D., Ph.D.<br>Pasi Antero Janne, M.D. Ph.D.<br>Bruce Evan Johnson, M.D.<br>David J. Kwiatkowski, M.D. Ph.D.<br>Geoffrey Liu, M.D.<br>Thomas Roberts, M.D.

Geoffrey Shapiro, M.D., Ph.D.
Arthur T. Skarin, M.D.
Jennifer S. Temel, M.D.
Thomas Lynch, M.D.

Northwestern University, Robert H. Lurie Comprehensive Cancer Center

Principal Investigator: Jyoti Patel, M.D.
Athanassios Argiris, M.D.
Andrew Evens, D.O.

Memorial Sloan-Kettering Cancer Center
1275 York Ave.
New York, N.Y. 10021

Table Of Contents

1.0 PROTOCOL SUMMARY AND/OR SCHEMA
2.0 OBJECTIVES AND SCIENTIFIC AIMS
3.0 BACKGROUND AND RATIONALE
4.0 STUDY DESIGN
5.0 THERAPEUTIC/DIAGNOSTIC AGENTS
6.1 PATIENT/SUBJECT INCLUSION CRITERIA
6.2 PATIENT/SUBJECT EXCLUSION CRITERIA
7.0 RECRUITMENT PLAN
8.0 PRETREATMENT EVALUATION
9.0 TREATMENT/INTERVENTION PLAN
10.0 EVALUATION DURING TREATMENT/INTERVENTION
11.0 TOXICITIES/SIDE EFFECTS
12.0 CRITERIA FOR THERAPEUTIC RESPONSE/OUTCOME ASSESSMENT
13.0 CRITERIA FOR REMOVAL FROM STUDY
14.0 BIOSTATISTICS
15.0 SUBJECT REGISTRATION
16.0 DATA MANAGEMENT ISSUES
16.1 QUALITY ASSURANCE
17.0 PROTECTION OF HUMAN SUBJECTS
18.0 INFORMED CONSENT PROCEDURES
19.0 REFERENCE(S)
20.0 APPENDICE(S) LIST APPENDICES HERE. APPENDICES WILL BE STORED IN A SEPARATE FILE AND WILL BE SUBMITTED IN ELECTRONIC AND/OR PAPER FORMAT. IF ELECTRONIC FORMAT, PLEASE SUBMIT ONE FILE PER APPENDIX.
APPENDIX A STUDY FLOWCHART..
APPENDIX B LUNG CANCER SYMPTOM SCALE (LCSS)...............
APPENDIX C KPS/ECOG PEROFRMANCE STATUS SCALE............
APPENDIX D RECIST CRITERIA..
APPENDIX E PATHOLOGY BLOCK AND SLIDE PROCESSING........
APPENDIX F THE CLINICAL RESPONSE OF PATIENTS WITH BRONCHIOLOALVEOLAR CARCINOMA TREATED WITH GEFITINIB
APPENDIX G SMOKING HISTORY QUESTIONNAIRE

1.0 PROTOCOL SUMMARY AND/OR SCHEMA

The primary objective of this phase II study is to determine the major objective response rate of OSI-774 in patients with unresectable or metastatic bronchioloalveolar cell variant of non-small cell lung cancer.

This will be a multi-institution phase II, open-label, study enrolling up to 100 patients. Enrollment will initially be limited to 50 patients. Enrollment beyond 50 patients will require approval from Genentech. Accrual to the trial will be temporarily halted until this is done.

All patients will receive OSI-774, 150 mg orally daily. The primary endpoint of this trial will be the major objective response rate (complete and partial responses). Toxicity, quality of life (as determined by the LCSS) and survival data will also be collected and measured.

Inclusion criteria:

- Pathologic evidence of either bronchioloalveolar cell carcinoma or a variant thereof after review by the reference MSKCC pathologist, Vanderbilt University pathologist, MD Anderson pathologist, Northwestern pathologist or Dana-Farber pathologist.

- Clinical stage IIIB (malignant pleural or pericardial effusion) or IV or recurrent/medically inoperable disease.

- Measurable or evaluable indicator lesions

- No prior or one chemotherapy regimen for NSCLC

- Three weeks since last chemotherapy, and three weeks since prior radiation therapy to a major bone-marrow containing area.

- ECOG $\leq$ 1 or Karnofsky performance status $\geq$ 80%

- Life expectancy $\geq$ 8 weeks

- Adequate hematologic, renal and hepatic function: WBC $\geq$ 3,000/ul, hemoglobin $\geq$ 9.0 g/dl, platelet count $\geq$ 100,000/ul, total bilirubin $\leq$ 1.0 mg/dl, AST $\leq$ 2.0 X UNL, creatinine $\leq$ 1.5 mg/dl or $Cl_{cr} \geq 55$ ml/min

- Use of an effective form of contraception (all men and women of child bearing potential)

- Signed informed consent

Exclusion criteria:

- Prior treatment with OSI-774 or other agents targeting the HER family (e.g.-ZD1839, cetuximab, trastuzumab, etc.)

- Two or more prior chemotherapy regimens

- Concurrent active cancer

- Uncontrolled central nervous system metastases (i.e. any known CNS lesion which is radiographically unstable, symptomatic and/or requiring escalating doses of corticosteroids)

- Pregnant or lactating women

- Malignancies within the past 5 years except for adequately treated carcinoma of the cervix or basal or squamous cell carcinomas of the skin

- Prior systemic cytotoxic chemotherapy for other malignant disease

- Significant medical history or unstable medical condition (unstable systemic disease: congestive heart failure, recent MI, unstable angina, active infection, uncontrolled hypertension).

Possible adverse reactions:

OSI-774: The primary toxicities consist of diarrhea, rash, nausea, vomiting, headache, and fatigue. The only dose-limiting toxicity observed to date is diarrhea. This event is dose-related and is generally controlled with the addition of loperamide therapy and treatment with OSI-774 doses of < 200 mg/day.

There appears to be a 3.5% (3/84 patients to date) incidence of Tarceva-induced lung disease/pneumonitis in patients with BAC similar to that observed with other anticancer drugs (e.g. gemcitabine, mitomycin, paclitaxel, G-CSF, etc.). The toxicity is manifested as an inflammation of the lungs that may be associated with increasing shortness of breath with exertion or at rest or increased coughing.

Pretreatment evaluation:

Prior to enrollment, patients will require complete medical history, physical examination and Karnofsky performance status determination within 2 weeks of study entry; laboratory evaluation including complete blood count, serum chemistry and electrolyte panel within 2 weeks of study entry; baseline ECG (electrocardiogram) within 2 weeks of

study entry; and appropriate medical imaging including CT scanning of all relevant disease sites within 4 weeks of study entry.

Following approximately 4 weeks of therapy, patients will undergo CT scanning to include all indicator lesions in order to detect a response to OSI-774 or progression of disease. CT scanning will be obtained again after 8 weeks of therapy and every 8 weeks thereafter. Physical examination and laboratory assessment, including complete blood count and chemistry panel will be required prior to initiating each cycle of chemotherapy. Patients will continue to receive OSI-774, unless they develop evidence of progressive disease, unacceptable toxicity, or any condition, which would pose undue risk to the patient through continuation.

2.0 OBJECTIVES AND SCIENTIFIC AIMS

- To determine the major objective response rate (p.r + c.r.) of OSI-774 in bronchioloalveolar carcinoma of the lung using a Fleming two-step trial design.

- To measure the quality of life in this population using the LCSS.

- To measure the duration of response and the time to disease progression in this population.

- To measure the median survival in this patient population.

3.0 BACKGROUND AND RATIONALE

Non-small cell lung cancer:

Lung cancer is the most lethal malignant tumor, killing over 150,000 Americans per year. [1] Three-quarters of patients have non-small cell lung cancer (NSCLC). Two-thirds of NSCLC patients have advanced disease (clinical stage IIIB/IV) and are considered incurable by surgery or radiation. The current standard of care for these patients is chemotherapy, which typically includes cisplatin or carboplatin in combination with one or two other active agents such as gemcitabine, vinorelbine, paclitaxel or docetaxel. [2] Meta-analyses have shown that cisplatin-based combination chemotherapy improves median survival by 2 months and one-year survival proportion by 10-15% in patients with advanced NSCLC. Therefore, the majority of patients with advanced NSCLC have disease, which is refractory to first-line chemotherapy, and those who respond initially eventually suffer progression of their disease when their cancer becomes resistant to the

first-line drugs. Historically, patients with progression of disease after first-line chemotherapy were either managed with best supportive care or enrolled in experimental drug trials. Recently, however, the results of two randomized, phase III trials suggest that treatment with docetaxel (Taxotere^{R}) should be considered the new standard of care for patients with good performance status and platinum-refractory disease.[3,4]

Bronchioloalveolar carcinoma:

Bronchiolo-alveolar carcinoma is a distinct subtype of adenocarcinoma of the lung that is increasing in incidence.[5] It particularly affects younger people and non-smokers, causing the loss of more years of productive life than the other types of lung cancer more associated with heavy tobacco abuse, such as SCLC and squamous cell NSCLC. It is defined as a malignant neoplasm of the lung that: 1) occurs in the absence of another primary adenocarcinoma; 2) has no central bronchogenic source; 3) is peripheral in location; 4) has an intact pulmonary interstitium; and 5) exhibits malignant cells growing along alveolar septae. It appears to arise from type 2 pneumocytes, grows along alveolar septa by lepidic ("scale-like") growth, and shows little if any desmoplastic or glandular change. BAC usually presents in three forms, a solitary peripheral nodule, multifocal disease, and a rapidly progressive pneumonic form, which appears to spread from lobe to lobe ultimately encompassing both lungs with little early evidence of distant metastases. The radiographic appearance of BAC is varied, with a single peripheral nodule or a diffuse infiltrate being the most common radiographic findings at presentation.[6] Patients with stages I and II BAC are treated like other NSCLC, but local (intrapulmonary) failures are common due to the superficial spread typical of this disease. Several reports suggest that Stage 3 and 4 BAC should be treated in a more supportive fashion, as these tumors are felt to be chemoresistant and radioresistant.[5] The diffuse multilobar involvement by thin sheets of tumor cells inside the airways makes BAC frequently impossible to resect completely, and local progression is frequently the life-limiting process in this disease. It is also very resistant to chemotherapy and incurable if not completely resectable.

OSI-774:

a.) Background:

OSI-774 (erlotinib; OSI-774), a quinazoline, is an orally active, potent, selective inhibitor of the EGFR (HER-1) tyrosine kinase. An overview of selected non-clinical and clinical information is presented here; complete details are available in the OSI-774 Investigator Brochure.

OSI-774 inhibits the human EGFR (HER-1) tyrosine kinase with an IC_{50} of 2 nM(0.786 mg/mL) in an in vitro enzyme assay and 20 nM (7.86 ng/mL) in intact tumor cells. This inhibition is selective for EGFR (HER-1) tyrosine kinase, results in cell cycle arrest at G_1, and is reversible. Oral administration of OSI-774 in mice results in a >70% reduction in EGFR (HER-1) autophosphorylation in human xenografts. Marked growth inhibition of HN5 and A431 xenografts in nude mice has been demonstrated. Data on drug exposure and antitumor responses in these xenograft models were analyzed to estimate the optimal plasma concentration of OSI-774 for antitumor activity in humans. Based on these models, a target plasma concentration of ≥ 500 ng/mL was selected.

b.) **Pharmacokinetics**:

The volume of distribution in both rats and dogs is ~3 L/kg. Oral bioavailability of an aqueous suspension is 77% in rats and 88% in dogs. OSI-774 is extensively metabolized in both rats and dogs, with only a small amount excreted unchanged in urine, bile, and feces. In vitro studies demonstrated that OSI-774 and its active metabolite OSI-420 are metabolized by CYP1A2, 3A4, 3A5, and/or 1A1. Plasma protein binding of OSI-774 ranges from 92% to 95% in mouse, rat, monkeys, and man and is 85% in dogs. In addition, OSI-774 plasma protein binding depends on the levels of α-1-acid glycoprotein (AAG). Thus, AAG might be a significant determinant of pharmacokinetic and possibly pharmacokinetic–pharmacodynamic relationships in patients.

c.) **Toxicology:**

The major effects attributed to OSI-774 in toxicology studies involved the hepatobiliary, gastrointestinal, and renal systems, as well as the cornea and skin, with reversibility noted on drug discontinuation. In an exploratory toxicology study performed in cynomolgus monkeys, emesis and loose stools were observed in animals treated at 100 mg/kg/day for 7 days. Elevations in serum bilirubin were noted in animals treated at 200 mg/kg/day for 7 days. One animal in the 200 mg/kg/day group expired. The cause of this death was not identified at necropsy. Dosing at 400 mg/kg/day was not tolerated beyond 4 days because of serum bilirubin elevations, frequent stools, decreased activity, and dehydration in 3 of 4 animals.

OSI-774 did not induce microbial or mammalian cell gene mutations in vitro and did not produce chromosomal aberrations in vitro or in vivo. No studies to assess the effects of OSI-774 on reproductive function or the potential for teratogenicity or carcinogenicity have been performed.

d.) **Summary of Phase I Findings:**

Phase I trials of OSI-774 have explored both schedule and dose to evaluate the safety, tolerability, and pharmacokinetic profile of the compound. Two Phase I trials in healthy

subjects and two Phase I trials in patients with advanced cancer have been completed. The primary toxicities consisted of diarrhea, rash, nausea, headache, emesis, and fatigue. The only dose-limiting toxicity was diarrhea. This event was dose related and was generally controlled with the addition of loperamide therapy and treatment with OSI-774 doses of <200 mg/day. The appearance of the rash seen in the clinical trials of OSI-774 conducted in healthy subjects and cancer patients has been similar. It was only loosely dose related and was seen commonly at doses of >25 mg/day. The rash was variable in onset, duration, and severity. The mechanistic basis of the rash remains uncertain; histopathologic examination of biopsies of the rash demonstrated polymorphonuclear leukocyte infiltration and mild epidermal hyperproliferation. In some cases, the rash improved despite continued dosing, and in general, it gradually resolved without sequelae following OSI-774 discontinuation. The rash did not result in study discontinuation in cancer patients in either of the Phase I trials.

Based on the ocular changes observed in the 12-month toxicology study in dogs, screening and follow-up ophthalmologic examinations were instituted in the Phase I and II trials in cancer patients. In the weekly dosing study (Study 248-005), the only reported OSI-774–related ocular event was an episode of mild watery eyes. In the daily dosing study (Study 248-004), 1 patient experienced moderate corneal edema/keratitis attributed to wearing contact lenses, although an influence of OSI-774 was not discounted. The event resolved with temporary discontinuation of both OSI-774 and contact lens use; there was no recurrence of symptoms with OSI-774 rechallenge in the absence of continued use of contact lenses.

e.) <u>Human Pharmacokinetic Studies</u>:

Review of the pharmacokinetic profiles from Studies 248-005 and 248-004 (see Investigator Brochure) revealed dose-related increases in exposure to OSI-774. Exposure to the active metabolite (OSI-420) represented ~10% of the parent compound, with an inter-patient variability in exposure of ~2-fold. Repetitive daily dosing resulted in drug accumulation. The target average plasma concentration for clinical efficacy (500 ng/mL) was achieved at doses of ≥100 mg in both the daily (Study 248-004) and weekly (Study 248-005) dosing studies. At the recommended dose of 150 mg/day, the accumulation ratio was 2.5±1.2, minimum plasma steady-state concentrations averaged 1.20±0.62 μg/mL, which is above the target concentration, and the half-life was 24.1 hours.

f.) <u>Phase II Trials: Studies in Patients with Advanced Cancer</u>:

The 150 mg/day dose of OSI-774 selected for all subsequent trials was based on pharmacokinetic parameters as well as the safety and tolerability profile of this dose level in Phase I trials in advanced, heavily pretreated cancer patients. Three Phase II trials of

the safety, tolerability, and antitumor activity of OSI-774 have been conducted in patients with advanced, refractory malignancies, including squamous cell carcinoma of the head and neck, ovarian carcinoma, and NSCLC. Patients in each of these studies received 150 mg/day of OSI-774. Dose reductions were allowed in the case of intolerance. Diarrhea was treated with loperamide therapy and/or dose reduction. Rash was treated with a variety of agents, including oral and topical antibiotics, corticosteroids, and other agents. Available preliminary data from all three Phase II trials demonstrate objective response rate of 10%–15%.

Non–Small Cell Lung Carcinoma. Study 248-1007 enrolled 56 patients at five centers with progressive, recurrent NSCLC previously treated with a platinum-based chemotherapy regimen. OSI-774 was administered at a daily dose of 150 mg. In this study, enrolled patients had measurable tumors that expressed a minimal level of EGFR (HER-1) as detected by immunohistochemical (IHC) analysis. All 56 patients were evaluable for antitumor response. Eight patients (14.3%) achieved an objective response (1 complete response, 7 partial responses; 6 were confirmed at Week 12 and beyond), 16 patients (28.6%) had stable disease lasting ≥12 weeks, and 28 patients (57.1%) had documented progression of their underlying malignancy. A relationship between response and the degree of EGFR (HER-1) over expression has not been established. Rash was observed in 80% of enrolled patients. The observed rash was mild in 46% of patients, moderate in 29%, and severe in 4%. Mild (Grade 1 or 2) diarrhea developed in 40% of patients and was moderate (Grade 3) in 3% of patients. Study drug was not discontinued for OSI-774–related adverse events in any of these patients, and only a single patient required transient dose reduction because of skin toxicity. Anecdotally, some of the most dramatic responses with OSI-774 and another agent of this class, ZD1839, have occurred in individuals with bronchioloalveolar carcinoma.[7 8]

4.0 STUDY DESIGN

Intervention and Treatment Plan:

This will be a phase II, open-label, multi-institution study enrolling up to 100 patients. The study will initially open at 2 sites (MSKCC and Vanderbilt). If sufficient responses are noted (see Statistical Section) to warrant additional accrual to more accurately define the response rate, additional sites will be opened to assure accrual to the planned 100 patients within one year from study inception. All patients will receive OSI-774. The primary endpoint of this trial will be the major objective response rate (complete and partial responses). Toxicity and survival data will also be determined. Molecular analysis of tumor tissue will be carried out to explore whether expression of specific proteins correlates with chemosensitivity or resistance. This analysis will include: the

construction of a BAC-specific tissue array (to include EGFR, phospho-EGFR, HER-2, HER-3, Akt, P-AKT), and sequencing of k-ras and PTEN.

5.0 THERAPEUTIC/DIAGNOSTIC AGENTS

Description: OSI-774 (erlotinib, Tarceva™) is supplied commercially by OSI Pharmaceuticals, Uniondale, NY.

Drug Accountability: All study drug required for completion of the study will be provided by OSI Pharmaceuticals, Inc. The recipient will acknowledge receipt of the drug by returning the INDRR-1 form indicating shipment content and condition to OSI Pharmaceuticals, Inc.. Damaged supplies will be returned. The investigator will be responsible for drug accountability.

Packaging and Storage: In addition to the active ingredient, OSI-774, tablets contain lactose, hydrous microcrystalline cellulose, sodium starch glycolate, sodium lauryl sulfate, and magnesium stearate. Study drug for daily oral administration will be supplied as 150 mg, 100 mg and 25 mg tablets of OSI-774 in bottles. Each bottle will contain 30 tablets. Study drug is stored at room temperature. For further details, see the OSI-774 Investigator Brochure.

Preparation and Administration: Tablets should be taken preferably in the morning 1 hour prior or 2 hours after a meal with up to 200 mL of water. Each bottle will contain 30 tablets, a quantity sufficient for 4 consecutive weeks of dosing, with overage. Patients who are unable to swallow tablets may dissolve the tablets in distilled water for administration.

Pretreatment Medications: None.

Warnings and Precautions: Based upon clinical experience to date, the following adverse effects may be associated with OSI-774 administration: The primary toxicities consist of diarrhea, rash, nausea, vomiting, headache, and fatigue. The only dose-limiting toxicity observed to date is diarrhea. This event is dose-related and is generally controlled with the addition of loperamide therapy and treatment with OSI-774 doses of < 200 mg/day.

6.1 PATIENT/SUBJECT INCLUSION CRITERIA

- Pathologic evidence of either bronchioloalveolar cell carcinoma or a variant thereof after review by the reference MSKCC pathologist, Vanderbilt University pathologist, MD Anderson pathologist, Northwestern pathologist or Dana-Farber pathologist.
- Clinical stage IIIB (malignant pleural or pericardial effusion) or IV or recurrent/medically inoperable disease
- Measurable or evaluable indicator lesions
- No prior or one chemotherapy regimen for NSCLC
- Three weeks since last chemotherapy, and three weeks since prior radiation therapy to a major bone-marrow containing area
- Karnofsky performance status ≥ 80% OR ECOG performance status ≤ 1
- Life expectancy ≥ 8 weeks
- Adequate hematologic, renal and/or hepatic function: WBC ≥ 3,000/ul, hemoglobin ≥ 9.0 g/dl, platelet count ≥ 100,000/ul, total bilirubin ≤ 1.0 mg/dl, AST ≤ 2.0 X UNL, creatinine ≤ 1.5 mg/dl or Clcr ≥ 55ml/min.
- Signed informed consent
- Effective contraception

6.2 PATIENT/SUBJECT EXCLUSION CRITERIA

- Prior exposure to OSI-774 or other treatments targeting the HER family axis (e.g.-trastuzumab, ZD1839, C225, etc.)
- Two or more prior chemotherapy regimens
- Concurrent active cancer
- Uncontrolled central nervous system metastases (i.e. any known CNS lesion which is radiographically unstable, symptomatic and/or requiring escalating doses of corticosteroids)
- Pregnant or lactating women
- Malignancies within the past 5 years except for adequately treated carcinoma of the cervix or basal or squamous cell carcinomas of the skin

- Prior systemic cytotoxic chemotherapy for other malignant disease
- Significant medical history or unstable medical condition (unstable systemic disease: congestive heart failure, recent MI, unstable angina, active infection, uncontrolled hypertension).

7.0 RECRUITMENT PLAN

Patients will be recruited through the outpatient clinics of the Memorial Sloan-Kettering Cancer Center and the Vanderbilt University/Inman Cancer Center. All patients will be under the care of attending medical oncologists of the MSKCC Thoracic Oncology Service or the thoracic oncologists of the other participating institution(s). Both men and women and members of all ethnic groups are eligible for this trial. The proposed study population is illustrated in the table below. Patients will be recruited from both centers and this will enhance ethnic and gender diversity.

Race/Ethnicity

**Gender**	White, not of Hispanic Origin	Black, not of Hispanic Origin	Hispanic	Asian or Pacific Islander	Unknown	Total
Male	40%	5%	5%	0	0	50%
Female	40%	5%	5%	0	0	50%
Total	80%	10%	10%	0	0	100%

8.0 PRETREATMENT EVALUATION

Pretreatment evaluation will include complete medical history, physical examination and Karnofsky performance status determination within 2 weeks of study entry; laboratory evaluation including complete blood count, serum chemistry (including total bilirubin, AST, alkaline phosphatase) and electrolyte panel within 2 weeks of study entry; baseline ECG within 2 weeks of study entry; completion of lung cancer symptom scale questionnaire; and appropriate baseline medical imaging including CT scanning of all relevant disease sites within 4 weeks of study entry.

9.0 TREATMENT/INTERVENTION PLAN

All patients will receive OSI-774, 150 mg orally daily. During the fifth and ninth weeks of OSI-774, patients will undergo CT scanning to include all indicator lesions in order to detect a response to OSI-774 or progression of disease. CT scanning will be obtained every 8 weeks thereafter. Physical examination and laboratory assessment, including complete blood count and chemistry panel will be required every 8 weeks.. Should drug toxicity develop, dose delays and/or dose reductions will be carried out according to criteria outlined in Section 11.0. Patients will continue receiving OSI-774 until they refuse further therapy, develop evidence of progressive disease, unacceptable toxicity, or any condition, which would, in the judgment of the investigator, affect assessments of clinical status to a significant degree or would pose undue risk to the patient through continuation.

10.0 EVALUATION DURING TREATMENT/INTERVENTION

Please see study flow chart (**Appendix A**)

During the first 4 weeks of OSI-774 therapy, patients will be seen on a weekly basis (+/- 3 working days if the patient is unable to be seen for logistical reasons such as holidays, etc). Thereafter, doctor visits and labs, including CBC, electrolyte and chemistry profile with liver function tests, will be every 4 weeks until completion of treatment. CT scanning of all indicator lesions will be performed every 8 weeks in order to determine response to OSI-774 or progression of disease. Patients will undergo repeat ECG at the end of treatment. The Lung Cancer Symptom Scale (LCSS) will be administered at baseline and every week for the first five weeks. After the ninth week, the LCSS will be administered every 4 weeks until the end of study. The LCSS patient form requires a second grade reading level and takes an average of 8 minutes to initially administer, including instruction time. Once a patient has become familiar with filling it out, it takes only 3-5 minutes to complete. The LCSS is designed as a disease- and site-specific measure of quality of life with lung malignancies and their effect on overall symptomatic distress, functional activities, and global quality of life.[9] The LCSS consists of two scales: one completed by the patient and an optional one for health care professionals ("counterpart observer") to provide context. Only the patient scale will be administered in this study. The nine-item patient scale includes nine visual analogue scales (100-mm horizontal line) where the patient puts a mark on a line to indicate intensity of response to the items in question (0 = lowest rating; 100 = highest rating). One LCSS sub-score is the average symptom burden index computed as the mean score for all six major symptoms (loss of appetite, fatigue, cough, dyspnea, hemoptysis, and pain).[9] Time to symptomatic progression will be measured in terms of a worsening in this index.

11.0 TOXICITIES/SIDE EFFECTS

NCI Common Toxicity Criteria (CTC): This study will utilize the Common Toxicity Criteria (CTC) version 2.0 for toxicity and Adverse Event reporting. A copy of the CTC version 2.0 can be downloaded from the CTEP home page (http://ctep.info.nih.gov). All treatment areas have access to a copy of the CTC version 2.0.

All toxic effects will be managed symptomatically, as indicated. Toxicities will be graded using the NCI Common Toxicity Criteria. Dose adjustments will be made as follows:

Dose reduction or interruption of study drug for adverse events may take place at any time during the study. Toxicity is based on NCI-CTC, Version 2.

Management of Grade 1 or 2 rash should include continuation of study drug at the current dose and symptomatic management. If skin rash persists or worsens over 10–14 days, dose reduction according to Table 2 should be considered. Management of Grade 3 rash should include dose reduction and symptomatic management. When skin toxicity improves by at least one grade level, the dose may be re-escalated as tolerated. In Phase II trials, this approach enabled dose re-escalation for the majority of patients requiring dose reduction for skin toxicity. Patients experiencing Grade 4 skin toxicity should be discontinued from the study.

For Grade 1 or 2 diarrhea, early intervention should include continuation of study drug at the current dose and initiation of loperamide therapy as described in **Table 3**. Persistent Grade 2 diarrhea, despite optimal medical management over 48–72 hours, should be managed by dose reduction according to **Table 2**. Patients should be maintained at the reduced dose without attempt at dose re-escalation. Patients experiencing Grade 3 diarrhea should interrupt study drug until resolution to Grade ≤1 and restart at a reduced dose according to **Table 2**. Patients experiencing Grade 4 diarrhea should be discontinued from the study.

Patients who experience Grade 3 fatigue should be managed with a dose reduction of 150mg to 100mg. Patients who experience Grade 4 fatigue should be discontinued from study.

Other serious adverse events or Grade 3 or 4 adverse events considered to be related to study drug should be managed with dose interruption until resolution of the event (Grade ≤1). Other serious adverse events or Grade 3 or 4 adverse events not resolving in 2 weeks will result in patient discontinuation. Patients may be rechallenged with study drug at the same dose level if the criteria for rechallenge are met. If the adverse event recurs after rechallenge, the patient should be withdrawn from the study.

Interstitial lung disease/pneumonitis associated with increasing shortness of breath with exertion or at rest may occur. Patients should be instructed to stop drug immediately if they note a worsening of baseline dyspnea or if they develop dyspnea while taking Tarceva. The attending physician should be instructed to immediately start steroids (1 mg/kg) once an infectious etiology has been satisfactorily excluded and to continue steroid treatment for several weeks with a gradual taper as appropriate to maintain optimal pulmonary function.

Table 2: OSI-774 Dose Level Reductions

Starting Dose	First Reduction	Second Reduction
150 mg/day	100 mg/day	50 mg/day

Within 2 weeks following a dose interruption or reduction, study drug–related toxicity must improve by at least one grade and be Grade ≤1, or further dose reduction by one level will be required. Dosing may be interrupted for a maximum of 2 weeks if clinically indicated and if the toxicity is not controlled by optimal supportive medication. No more than two dose reductions will be allowed.

Table 3 outlines study drug dose modification criteria for study drug–related toxicities as well as guidelines for their management.

Table 3: Dosage Modification Criteria and Guidelines for Management of Study Drug–Related Toxicities

Toxicity, NCI-CTC Grade	Study Drug Dose Modification	Guideline for Management
**Diarrhea**		
Grade 1	None	Consider loperamide (4 mg at first onset, followed by 2 mg q 2–4 hours until diarrhea free for 12 hours)
Grade 2	None	Loperamide (4 mg at first onset, followed by 2 mg q 2–4 hours until diarrhea free for 12 hours)
Grade 3	Interrupt	Interrupt until resolution to Grade ≤1, and restart at reduced dose
Grade 4	Discontinue study	
**Rash**		
Grade 1	None	Any of the following: minocycline[a], topical tetracycline, topical clindamycin, topical silver sulfadiazine, diphenhydramine, oral prednisone (short course) at discretion of investigator
Grade 2	None	Manage as described above
Grade 3 (or intolerable Grade 2)	Dose reduction; dose can be re-escalated when rash is Grade ≤2	Manage as described above
Grade 4	Discontinue study	Manage as described above

[a] Recommended dose: 200 mg po bid (loading dose) followed by 100 mg po bid for 7–10 days.

Investigators are required to report any SERIOUS adverse event that may be expected or unexpected, as defined below, and reasonably or probably regarded as caused by investigational product occurring within a protocol-defined period of treatment and post-treatment follow-up.

SAFETY REPORTING OF ADVERSE EVENTS

Adverse Event and Reporting Definitions:

(For Memorial Sloan-Kettering Cancer Center and Vanderbilt-Ingram Cancer Center)

In the event of an adverse event, the first concern will be for the safety of the subject. Investigators are required to report to Genentech Drug Safety any serious adverse event, whether expected or unexpected, and which is assessed by the investigator to be reasonably or possibly related to or caused by Tarceva.

All events meeting these criteria will be reported for the time period beginning with any amount of exposure to Tarceva through the protocol-defined follow-up period. Serious criteria, definitions, and guidance for reporting follow.

An adverse event (AE) is any untoward medical occurrence in a subject participating in an investigational study or protocol regardless of causality assessment. An adverse event can be an unfavorable and unintended sign (including an abnormal laboratory finding), symptom, syndrome or disease associated with or occurring during the use of an investigational product whether or not considered related to the investigational product.

Serious adverse events (SAE) are adverse events occurring at any dose which meet one or more of the following serious criteria:

Results in death (i.e. the AE caused or lead to death)

Is life-threatening (i.e. the AE placed the subject at immediate risk of death; it does not apply to an AE which hypothetically might have caused the death if it were more severe)

Requires or prolongs inpatient hospitalization (i.e. the AE required at least a 24-hour inpatient hospitalization or prolonged a hospitalization beyond the expected length of stay; hospitalizations for elective medical/surgical procedures, scheduled treatments, or routine check-ups are not SAEs by this criterion)

Is disabling (i.e. the AE resulted in a substantial disruption of the subject's ability to carry out normal life functions)

Is a congenital anomaly/birth defect (i.e., an adverse outcome in a child or fetus of a subject exposed to the study drug prior to conception or during pregnancy)

It does not meet any of the above serious criteria but may jeopardize the subject and may require medical or surgical intervention to prevent one of the outcomes listed above

Expected adverse events are those adverse events that are listed or characterized in the current Investigator Brochure.

Unexpected adverse events are those not listed in the current Investigator Brochure (I.B.) or not identified. This includes adverse events for which the specificity or severity is not consistent with the description in the I.B. For example, under this definition, hepatic necrosis would be unexpected if the I.B. only referred to elevated hepatic enzymes or hepatitis.

Reporting of Serious Adverse Events Associated with Tarceva- (Memorial Sloan-Kettering Cancer only)

All SAEs that are serious and reasonably or probably related to Tarceva (this applies to both expected and unexpected events) should be recorded on a MedWatch 3500 Form and faxed to:

Genentech Drug Safety
Tel: Linda Hartmann, Drug Safety Scientist
(650) 225-5051

-OR-
Rich Brotherton, Clinical Safety Associate
(650) 225-4562
Fax: (650) 225-4682 or (650) 225-4683
(Please use the safety reporting fax cover sheet attached to this document for your fax transmission)

AND:
Dr. David Johnson
C/ O Teresa Patterson, CCRC
Clinical Trials Specialist
Vanderbilt-Ingram Cancer Center
491 Preston Research Building
Nashville, TN 37232-6868
Telephone: 615-936-5874
Fax: 615-936-5850.

AND:

Memorial Sloan Kettering Cancer Center IRB Office

MedWatch 3500 Reporting Guidelines:

In addition to completing appropriate patient demographic and suspect medication information, the report should include the following information within the Event Description (section 5) of the MedWatch 3500 form:

Protocol description (and number, if assigned)
Description of event, severity, treatment, and outcome, if known
Supportive laboratory results and diagnostics
Investigator's assessment of the relationship of the adverse event to each investigational product and suspect medication

Follow-up information:

- Additional information may be added to a previously submitted report by any of the following methods:

- Adding to the original MedWatch 3500 report and submitting it as follow-up.

- Adding supplemental summary information and submitting it as follow-up with the original MedWatch 3500 form.

- Summarizing new information and faxing it with a cover letter including subject identifiers (i.e. D.O.B. initial, subject number), protocol description and number, if assigned, brief adverse event description, and notation that additional or follow-up information is being submitted (The subject identifiers are important so that the new information is added to the correct initial report)

Occasionally Genentech may contact the reporter for additional information, clarification, or current status of the subject for whom and adverse event was reported. For questions regarding SAE reporting, you may contact the Genentech Drug Safety representative noted above.

Study Drug Relationship:

The investigator will determine which events are associated with the use of study drug. For reporting purposes, an AE should be regarded as possibly related to the use of the investigational product if the investigator believes:

- There is a clinically plausible time sequence between onset of the AE and Tarceva administration; and/or

- There is a biologically plausible mechanism for Tarceva causing or contributing to the AE.

- The AE cannot be attributed solely to concurrent/underlying illness, other drugs, or procedures.

Reporting Requirements for IND Holders (Vanderbilt-Ingram Cancer Center only)

a. Expedited IND Safety Reports:

For Investigator Sponsored IND Studies, some additional reporting requirements for the FDA apply in accordance with the guidance set forth in 21 CFR § 312.32.

Events meeting the following criteria need to be submitted to the Food and Drug Administration (FDA) as expedited IND Safety Reports according to the following guidance and timelines:

***7 Calendar-Day Telephone or Fax Report*:**

The Sponsor-Investigator is required to notify the FDA of any fatal or life-threatening adverse event that is unexpected and assessed by the investigator to be possibly related to the use of Tarceva. An unexpected adverse event is one that is not already described in the Investigator Brochure. Such reports are to be telephoned or faxed to the FDA and Genentech within 7 calendar days of first learning of the event. Each telephone call or fax transmission (see fax number below) should be directed to the FDA new drug review division in the Center for Drug Evaluation and Research or in the product review division for the Center for Biologics Evaluation and Research, whichever is responsible for the review of the IND.

15 Calendar-Day Written Report:

The Sponsor-Investigator is also required to notify the FDA and all participating investigators, in a written IND Safety Report, of any serious, unexpected AE that is considered reasonably or possibly related to the use of Tarceva. An unexpected adverse event is one that is not already described in the Investigator Brochure.

Written IND Safety Reports should include an Analysis of Similar Events in accordance with regulation 21 CFR § 312.32. All safety reports previously filed with the IND concerning similar events should be analyzed. The new report should contain comments on the significance of the new event in light of the previous, similar reports.

Written IND safety reports with Analysis of Similar Events are to be submitted to the FDA, Genentech Drug Safety, and all participating investigators within 15 calendar days of first learning of the event. The FDA prefers these reports on a MedWatch 3500 Form but alternative formats are acceptable (e.g. summary letter).
FDA fax number for IND Safety Reports:

1 (800) FDA 1078

All written IND Safety Reports submitted to the FDA by the Sponsor-Investigator must also be faxed to: Genentech Drug Safety

Fax: (650) 225-4682 or (650) 225-4683(Please use the safety reporting fax cover sheet attached to this document for your fax transmission)

AND:

Dr. David Johnson
C/ O Teresa Patterson, CCRP
Clinical Trials Specialist
Vanderbilt-Ingram Cancer Center
491 Preston Research Building
Nashville, TN 37232-6868
Telephone: 615-936-5874
Fax: 615-936-5850.

AND:

Vanderbilt-Ingram Cancer Center IRB Office

For questions related to safety reporting, contact:

Genentech Adverse Events
Tel: Linda Hartmann, Drug Safety Scientist
(650) 225-5051
-OR-
Rich Brotherton, Clinical Safety Associate
(650) 225-4562
Fax: (650) 225-4682 or (650) 225-4683
(Please use the safety reporting fax cover sheet attached to this document for your fax transmission)

b. IND Annual Reports

In accordance with the regulation 21 CFR § 312.32, the Sponsor-Investigator shall within 60 days of the anniversary date that the IND went into effect submit a brief report of the progress of the investigation. Please refer to Code of Federal Regulations, 21 CFR § 312.32 for a list of the elements required for the annual report. All IND annual reports submitted to the FDA by the Sponsor-Investigator should be copied to Genentech. Copies of such reports should be mailed to:

Genentech, Inc.
ATTN: Stephanie Royer
Tarceva IST Coordinator
1 DNA Way, Mailstop #59
South San Francisco, CA 94080-4990
Tel: (650) 225-6698

Retention of Records

U.S. FDA regulations (21 CFR 312.62[c]) require that records and documents pertaining to the conduct of this study and the distribution of investigational drug, including CRFs, consent forms, laboratory test results, and medication inventory records, must be retained by the Principal Investigator for 2 years after marketing application approval. If no application is filed, these records must be kept 2 years after the investigation is discontinued and the U.S. FDA and the applicable local health authorities are notified.

12.0 CRITERIA FOR THERAPEUTIC RESPONSE/OUT COME ASSESSMENT

For the purposes of this study, patients will undergo repeat CT scanning of all indicator lesions following four and eight weeks of OSI-774 therapy. Thereafter, CT scanning will be obtained every eight weeks. See Appendix F for methods of response assessment and classification.

13.0 CRITERIA FOR REMOVAL FROM STUDY

If at any time the patient develops progressive disease he/she will be taken off study and referred for alternative therapy.

If at any time the patient develops unacceptable toxicity he/she will be removed from study.

If at any time the patient is found to be ineligible for the protocol as designated in the section on Criteria for Patient/Subject Eligibility (i.e., a change in diagnosis), the patient will be removed from the study.

Administration of radiotherapy or of any other chemotherapeutic drug during the trial will result in the patient being taken off study.

The patient will be taken off study if he/she withdraws consent.If at any time the patient develops any condition that would, in the judgment of the investigator, affect assessments of clinical status to a significant degree or would pose undue risk to the patient through continuation, the patient will be removed from the study.

14.0 BIOSTATISTICS

This will be a multicenter non-randomized single arm phase II trial seeking to determine whether OSI-774 results in a meaningful proportion of responses accompanied by symptomatic relief and improved quality of life in patients for whom no other standard therapy is available or commonly effective.

Patients will be asked to complete the Lung Cancer Symptom Scale at baseline and during the trial. Changes in the mean 100 mm visual analogue scale scores measuring disease-related symptoms for patients will be recorded weekly for the first four weeks of treatment and monthly thereafter. Nine symptoms (hemoptysis, pain, appetite, cough, dyspnea, activity, quality of life, fatigue, and lung cancer symptoms) will be recorded. Each patient's score on a given symptom will undergo square-root transformation to stabilize variance, followed by calculation of the difference from baseline to weeks 2, 3, 4, 5 and "best" timepoint. We have previously reported disease-related symptom improvement with this method [Miller, Cancer, 75(4), 1995].

A response rate of 15% or greater would be of interest in this patient population. An objective response rate of ≥ 25%, in conjunction with significant improvements in the LCSS may warrant consideration for regulatory submission. A complete response rate ≥ 10% would also warrant further study. A 2-stage design will be utilized. At the first stage, a total of 21 patients will be enrolled. If ≥ 1 response is noted, an additional 29 patients will be recruited to the second stage. This design assures that if no response is observed in the first 21 patients the likelihood of the true response rate being > 15% is < 5%. If ≥ 12 patients in the total sample of 50 patients demonstrate an objective response or ≥ 5 patients demonstrate a complete response, the sample size will be increased to 100 patients so as to provide better precision around the objective response rate and to generate additional safety and QOL data via the LCSS.

15.0 Subject Registration and Randomization Procedures

15.1 PATIENT/SUBJECT REGISTRATION

The following person(s) can obtain informed consent:

Mark G. Kris, M.D.
Lee M. Krug, M.D.
Vincent A. Miller, M.D.
Herbert Oettgen, M.D.
Jorge E. Gomez, M.D.
William Pao, M.D.

Christopher G. Azzoli, M.D.
Naiyer Rizvi, M.D.

Confirm with electronic medical record that the patient has signed the Notice of Privacy Practice. This must be obtained before the eligibility confirmation and obtaining of the research informed consent.

Confirm eligibility as defined in the section entitled Criteria for Patient/Subject Eligibility.

Obtain written informed consent, by following procedures defined in section entitled Informed Consent Procedures.

All patients must be registered through the Department of Medicine's (CTO) registration system at Memorial Sloan-Kettering Cancer Center. The CTO registry is available Monday through Friday from 8:15am - 5:00pm EST at 123-2150 (646/227-2150 from outside MSKCC). The last page of the signed consent form, the signature page of the Research Authorization, and a completed Eligibility Checklist must be faxed to the CTO registry at the time of registration. The fax number is 212-557-0786.

During the telephone registration process registerring individuals will be required to answer specific eligibility questions and provide the following information:

Registering Individual	[Last, First Name]
Notice of Privacy Status	[Yes, No]
Research Authorization	[Date]
MSKCC IRB Protocol#	
Attending of Record (if applicable)	[Last, First Name]
Consenting Professional	[Last, First Name]
Informed Consent Date	
Patient's Full Name	[Last, First Name]
Patient MRN	

For Vanderbilt-Ingram Cancer Center, University of Texas, MD Anderson, Dana Farber Cancer Institute and Northwestern University, Robert H. Lurie Comprehensive Cancer Center patients:

To register and randomize a patient, the investigator will call the Vanderbilt University, Clinical Trials Office at (615) 322-4967 and ask that Norma Campbell, RN or the person covering for her be paged. The following information will be requested:

Investigator Identification

Institution name and/or affiliate
Investigator's name
Patient's Identification
Patient's name or initials and chart number
Patient's Social Security number
Eligibility Verification

Patients must meet all of the eligibility requirements listed in the sponsor's protocol.

Cancellation Guidelines
If a patient does not receive any protocol therapy, the patient may be canceled. Reasons for cancellation should be submitted in writing as soon as possible.

The following persons may obtain informed consent:

David Johnson, M.D.
Alan Sandler, M.D.
Jeffrey Sosman, M.D.
Kenneth Hande, M.D.
Kenneth Wyman, M.D.
Thao Dang, M.D.
David Carbone, Ph.D.,M.D.
Roy S. Herbst, M.D., Ph.D.
Anne S Tsao, M.D.
George Blumenchein, Jr. , M.D.
Bonnie Glisson, M.D.
Frank Fossella, M.D.
Waun Ki Hong, M.D.
Charles Lu, M.D.
Yun Oh, M.D.
Vassiliki Papadimitrakopoulou, M.D.
Kathering Pisters, M.D.
Hai Tran, Pharm. D.
Ralph Zinner, M.D.
Jonathan Kurie, M.D.
Merril Kies, M.D.
Edward Kim, M.D.
Jyoti Patel, M.D.
Athanassios Argiris, M.D.
Andrew Evens, D.O.

Panos Fidias, M.D.
Sarada Gurubhagavatula, M.D.
John Heymach, M.D., Ph.D.
Pasi Antero Janne, M.D. Ph.D.
Bruce Evan Johnson, M.D.
David J. Kwiatkowski, M.D. Ph.D.
Geoffrey Liu, M.D.
Thomas Roberts, M.D.
Geoffrey Shapiro, M.D., Ph.D.
Arthur T. Skarin, M.D.
Jennifer S. Temel, M.D.
Thomas Lynch, M.D.

15.2 RANDOMIZATION NOT APPLICABLE.

16.0 DATA MANAGEMENT ISSUES

A Research Study Assistant (RSA) will be assigned to the study. The responsibilities of the RSA include project compliance, data collection, abstraction and entry, data reporting, regulatory monitoring, problem resolution and prioritization, and coordinate the activities of the protocol study team. The data collected for this study will be entered into a secure database. Source documentation will be available to support the computerized patient record.

16.1 QUALITY ASSURANCE

Weekly registration reports will be generated to monitor patient accruals and completeness of registration data. Routine data quality reports will be generated to assess missing data and inconsistencies. Accrual rates and extent and accuracy of evaluations and follow-up will be monitored periodically throughout the study period and potential problems will be brought to the attention of the study team for discussion and action

Random-sample data quality and protocol compliance audits will be conducted by the study team, at a minimum of two times per year, more frequently if indicated.

17.0 PROTECTION OF HUMAN SUBJECTS

Every effort will be made to maintain patient confidentiality. Research and hospital records are confidential. Patient's names or any other personally identifying information will not be used in reports or publications resulting from this study. The Food and Drug Administration, other

authorized agencies (e.g., qualified monitors from MSKCC or the NCI etc.), and appropriate personnel may review patient records as required.

PRIVACY

It is the responsibility of the Research Staff to ensure that Memorial Sloan-Kettering Cancer Center has on file a written acknowledgment of receipt by the subject of the Center's Notice of Privacy Practices. If the subject has not already done so, he/she must sign such an acknowledgment before participating in this study.

MSKCC's Privacy Office may allow the use and disclosure of protected health information pursuant to a completed and signed Research Authorization form. The use and disclosure of protected health information will be limited to the individuals described in the Research Authorization form. A Research Authorization form must be completed by the Principal Investigator and approved by the IRB and Privacy Board.

INCLUSION OF CHILDREN IN RESEARCH

This protocol/project does not include children because the number of children is limited and because the majority is already accessed by a nationwide pediatric cancer research network. This statement is based on exclusion 4b of the NIH Policy and Guidelines on the Inclusion of Children as Participants in Research Involving Human Subjects.

Risks: It is not known whether treatment with OSI-774 will be more or less harmful than conventional chemotherapy.

Benefits: It is hoped OSI-774 will improve response rates, and/or improve survival when compared to other chemotherapy regimens use din this setting.

Possible toxicities/side effects:

Possible toxicities/side effects of OSI-774 include:

a. Acneiform Rash

an acne-like skin rash was observed in >75% of patients treated in three Phase II trials using a dosing regimen to this study. The rash was generally mild to moderate in severity. Severe rash occurred in ~2%-10% of patients. In the Phase II NSCLC trial (Study 248-1007), 4% of patients experienced Grade 3 rash, but none required dose reduction. The rash appeared to be treatable in some patients with standard acne therapies, including topical and oral antibiotics.

b. Diarrhea

Dose-limiting diarrhea was observed in clinical trials using OSI-774 doses of > 150 mg/day. In patients treated with OSI-774 150 mg/day, diarrhea was less common and was characterized as two to three loose stools per day. In the Phase II NSCLC trial (Study 248-1007), 1 patient experienced Grade 3 diarrhea and was treated with loperamide; the OSI-774 dose was not reduced. The frequency and severity of diarrhea rarely hindered OSI-774 administration and could be managed with loperamide.

c. Other Safety Concerns

Other less common adverse events observed in previous clinical trials of single agent OSI-774 include headache, nausea and vomiting, and fatigue. Ocular toxicity was seen in nonclinical animal models. A single patient in a previous clinical trial experienced a serious adverse event of corneal abrasion/keratitis while wearing contact lenses. A single patient with 9-year history of HIV infection developed pancreatitis 32 weeks after starting OSI-774. This was associated with a change in anti-retroviral therapy; however, the investigator considered the event to be possibly related to OSI-774. Approximately 4% of patients in the single-agent, Phase I and II trials developed liver function abnormalities.

Because of the potential for drug-drug interaction between OSI-774 an warfarin, patients in this study who are receiving concomitant warfarin therapy will have INR results obtained at screening and during treatment as follows: patients will have INR results checked weekly during the first four weeks of treatment. Thereafter, patients will have INR results obtained monthly.

Patients will be carefully monitored during the entire treatment and follow-up periods.

Consent process: Participation in this trial is voluntary. All patients will be required to sign a statement of informed consent that must conform to MSKCC IRB guidelines.

Costs: Patients will be charged for physician visits, routine laboratory and radiologic studies required for monitoring their condition. Patients will be charged for the necessary pathologic services required to render a diagnosis of cancer. They will not be charged for correlative pathologic testing. Patients will not be billed for the pharmacy cost of the chemotherapeutic drugs administered.

Alternatives: Alternative treatment options include observation, standard therapy, or participation in other investigational studies.

Confidentiality: Every effort will be made to maintain patient confidentiality. Research and hospital records are confidential. Patient's names or any other personally identifying information will not be used in reports or publications resulting from this study. The

Food and Drug Administration, other authorized agencies (e.g., qualified monitors from MSKCC or the NCI etc.), and appropriate personnel may review patient records as required.

18.0 INFORMED CONSENT PROCEDURES

All patients will be required to sign a statement of Informed Consent, which meets the requirements of the Code of Federal Regulations 21 CFR 50.25 (Elements of Informed Consent) and the IRB of this center.

It is the responsibility of the medical oncologist to describe the nature of this study to the patient. Once the purpose, methods, risks, and benefits of the study have been explained to the patient in a satisfactory manner, including a review of the Informed Consent document, three copies of the Informed Consent shall be signed and dated by both patient (or his or her guardian) and physician. One copy will be returned to the patient, one copy will be placed in the patient's medical record, and the third will be delivered to the MSKCC Clinical Trials Office.

RESEARCH AUTHORIZATION

Procedures for obtaining Research Authorization: Before any protocol-specific procedures are carried out, investigators and/or designated staff will fully explain the details of the protocol, study procedures, and the aspects of patient privacy concerning research specific information. In addition to signing the IRB Informed Consent, all patients must sign the Research Authorization component of the informed consent form. The Research Authorization requires a separate signature from the patient. The original signed documents will become part of the patient's medical record, and each patient will receive a copy of the signed documents.

19.0 REFERENCE(S)

1. Greenlee R, Hill-Harmon M, Murray T, Thun M. Cancer Statistics, 2001. CA Cancer J Clin 2001; 51:15-36.
2. Schiller JH, Harrington D, Sandler A, et al. A randomized phase III trial of four chemotherapy regimens in advanced non-small cell lung cancer. Proc Am Soc Clin Oncol 2000 (abstr 2); 19:1a.
3. Fossella FV, DeVore R, Kerr RN, et al. Randomized phase III trial of docetaxel versus vinorelbine or ifosfamide in patients with advanced non-small-cell lung cancer previously treated with platinum-containing chemotherapy regimens. The TAX 320 Non-Small Cell Lung Cancer Study Group. J Clin Oncol 2000; 18:2354-62.
4. Shepherd FA, Dancey J, Ramlau R, et al. Prospective randomized trial of docetaxel versus best supportive care in patients with non-small-cell lung cancer previously treated with platinum-based chemotherapy. J Clin Oncol 2000; 18:2095-103.
5. Barkley JE, Green MR. Bronchioloalveolar carcinoma. J Clin Oncol 1996; 14:2377-86.
6. Akata S, Fukushima A, Kakizaki D. CT scanning of bronchioloalveolar carcinoma, specific appearances. Lung Cancer 1995; 12:221-30.
7. Miller V, Johnson D, Heelan R, et al. A Pilot Trial Demonstrates the Safety of ZD1839 ('Iressa'), an Oral Epidermal Growth Factor Receptor Tyrosine Kinase Inhibitor (EGFR-TKI), in Combination with Carboplatin (C) and Paclitaxel (P) in Previously Untreated Advanced Non-Small Cell Lung Cancer (NSCLC), Proc Am Soc Clin Oncol, San Francisco, CA, 2001. Vol. 20.
8. Negoro S, Nakagawa K, Fukuoka M, et al. Final Results of a Phase I Intermittent Dose-EscalationTrial of ZD1839 ('Iressa') In Japanese Patients With Various Solid Tumours, Proc Am Soc Clin Oncol, San Francisco, CA, 2001. Vol. 20.
9. Hollen PJ, Gralla RJ, Kris MG, Cox C. Quality of life during clinical trials: conceptual model for the lung cancer symptom scale (LCSS). Support Care Cancer 1994; 2:213-22.
