# Supplementary material for: KRAS Mutations and Primary Resistance of Lung Adenocarcinomas to Gefitinib or Erlotinib
Source: PLoS Med. 2005 Jan 25;2(1):e17. doi: 10.1371/journal.pmed.0020017 (PMC545207; doi:10.1371/journal.pmed.0020017)
Supplement: Protocol S3 — (60 KB PDF). [file pmed.0020017.sd003.pdf]

MEMORIAL SLOAN-KETTERING CANCER CENTER
ESTABLISHED 1884

Institutional Review Board/Privacy Board

TO: Vincent Miller, MD
Department of Medicine

FROM: Roger S. Wilson, MD
Chairman, IRB/Privacy Board

DATE: June 1st, 2004

RE: Request for Waiver of Authorization, WA0199-04

Your request for a Waiver of Authorization entitled "Molecular Determinants of Sensitivity and Resistance to Gefitinib" was reviewed by the Privacy Board and was ***approved.***

The Waiver of Authorization is approved only for the reasons set out in your request. The approval number is WA0199-04. Please remember that you, the Principal Investigator, are responsible for tracking the disclosure of PHI under this waiver. Before you disclose any information outside the institution, the data set needs to be reviewed and approved by the Privacy Office or its representatives. Please E-mail the data set to hipaares@mskcc.org. If you have any questions, please call the HIPAA Research Help Line (646-735-8048). In addition, if you would like to review the same PHI for a different research question, another waiver of authorization must be submitted to the Privacy Board.

Institutional Review Board/Privacy Board

TO: Dr. V.A. Miller

FROM: Dr. Roger S. Wilson
Chairman, Institutional Review Board/Privacy Board

DATE: February 25, 2004

RE: Protocol and Consent Form Amendments for Protocol #92-055A(12)

Your protocol and consent form amendments for Protocol #92-055A(12) entitled "Preclinical Studies of Blood, Urine, Bone Marrow, and Tissues Collected from Patients with Lung Cancer and Mesothelioma", as outlined in your attached memo, were reviewed at the February 24, 2004 Institutional Review Board/Privacy Board meeting and were approved.

RSW: ac
Enclosure

Memorial Sloan-Kettering Cancer Center
1275 York Avenue, New York, New York 10021
Telephone 212.639.7592

NCI-designated Comprehensive Cancer Center

Institutional Review Board/Privacy Board

TO: Dr. Vincent Miller

FROM: Dr. Roger S. Wilson
Chairman, Institutional Review Board/Privacy Board

DATE: July 14, 2004

RE: Protocol and Consent Form Amendments for Protocol # 02-010A(9)

Your protocol and consent form amendments for Protocol # 02-010A(9) entitled "Multicenter Phase II Trial of OSI-774 (Erlotinib, Tarceva^{TM}) in Patients with Advanced Bronchioloalveolar Cell Lung Cancer", as outlined in your attached memo, were reviewed at the July 13, 2004 Institutional Review Board meeting and were approved.

RSW:ch
Enclosure

Memorial Sloan-Kettering Cancer Center
1275 York Avenue, New York, New York 10021
Telephone 212 639 7592

NCI-designated Comprehensive Cancer Center
